# A Gas Chromatographic System for the Detection of Ethylene Gas Using Ambient Air as a Carrier Gas

**DOI:** 10.3390/s17102283

**Published:** 2017-10-07

**Authors:** Nayyer Abbas Zaidi, Muhammad Waseem Tahir, Michael J. Vellekoop, Walter Lang

**Affiliations:** 1 International Graduate School for Dynamics in Logistics (IGS), University of Bremen, 28359 Bremen, Germany; wtahir@imsas.uni-bremen.de (M.W.T.); wlang@imsas.uni-bremen.de (W.L.); 2 Institute for Microsensors, -Actuators and -Systems (IMSAS), University of Bremen, 28334 Bremen, Germany; mvellekoop@imsas.uni-bremen.de

**Keywords:** gas chromatographic system, electrochemical ethylene gas sensor, preconcentrator, 3D printed gas chromatographic column

## Abstract

Ethylene gas is a naturally occurring gas that has an influence on the shelf life of fruit during their transportation in cargo ships. An unintentional exposure of ethylene gas during transportation results in a loss of fruit. A gas chromatographic system is presented here for the detection of ethylene gas. The gas chromatographic system was assembled using a preconcentrator, a printed 3D printed gas chromatographic column, a humidity sensor, solenoid valves, and an electrochemical ethylene gas sensor. Ambient air was used as a carrier gas in the gas chromatographic system. The flow rate was fixed to 10 sccm. It was generated through a mini-pump connected in series with a mass flow controller. The metal oxide gas sensor is discussed with its limitation in ambient air. The results show the chromatogram obtained from metal oxide gas sensor has low stability, drifts, and has uncertain peaks, while the chromatogram from the electrochemical sensor is stable and precise. Furthermore, ethylene gas measurements at higher ppb concentration and at lower ppb concentration were demonstrated with the electrochemical ethylene gas sensor. The system separates ethylene gas and humidity. The chromatograms obtained from the system are stable, and the results are 1.2% repeatable in five similar measurements. The statistical calculation of the gas chromatographic system shows that a concentration of 2.3 ppb of ethylene gas can be detected through this system.

## 1. Introduction

The loss of food during the transportation, production, and selling are beyond expectations. A survey reported by Gustavsson et al. reveals that one-third of the food produced around the world is lost. A large proportion of food damage is linked with food loss during transportation in cargo ships. These losses occur due to insufficient atmosphere monitoring in cargo containers. China alone lost billions of RMB per annum during food transportation [1,2]. 

To increase the shelf life after the fruit harvest, key factors are low temperature and maintained carbon dioxide, humidity, and ethylene gas in the container. Of these, temperature has the greatest influence on the increase in the shelf life of fruit. In the case of strawberries, a small temperature variation of a few degrees for an hour results in a decrease in the shelf life by a day. Fruit respirates, consumes oxygen, and produces carbon dioxide and ethylene gas. To maintain the quality of fruit during transportation, these parameters need to be measured. Control of these parameters can help regulate the rate of respiration and metabolism in fruit and thus help retain freshness in transportation containers. Measuring carbon dioxide, humidity, and temperature is relatively straightforward, and commercially sensors are available to detect them. However, to develop a system that detects ethylene gas in ppb showing no cross-sensitivity toward carbon dioxide is still a challenge [3,4,5]. 

Ethylene is a gas produced from fruit during their ripeness. It is an invisible and fragrance-free gas efflux from fruit during their maturity. For instance, at 20 °C, 1 kg of kiwi produces 1 μL of ethylene per hour. Ethylene gas at a concentration of 1 ppm is sufficient enough to trigger the ripening process in climacteric fruit. Ethylene, being lightweight (0.97–0.99 kg·m^−3^) like air, can diffuse easily in neighboring commodities [6,7]. 

The measurement of ethylene in a storage container is essential for two reasons. The first is to know the current ripening state of the fruit and the second is to predict its remaining shelf life using their ripening models. If, by any chance, the concentration of ethylene reaches 1 ppm, then vents in the container become active, which allows fresh air in and decreases ethylene concentration [3].

Existing methods used for the detection of ethylene gas are photoacoustic spectroscopy, metal-oxide semiconductor-based sensors, electrochemical sensors, electro-catalytic sensors, and nondispersive spectroscopy; see [3,8] for a review. All techniques are currently either too expensive or do not have a high sensitivity in the presence of humidity. In 2016, Biasio et al. used non-dispersive infrared spectroscopy, NDIR for the detection of ethylene gas and the system detects ethylene concentration of 20 ppm [9]. Krivec et al. in 2015 used two types of chemoresistive MOX sensor structure, based on SnO_2_ and WO_3_, to measure ethylene at concentrations of 200 ppm [10]. In 2017, Besar et al. used an organic field effect transistor for the detection of ethylene gas with a detection limit of 25 ppm [11]. The detection of the sub-ppb level is still under study. 

Work in the field of detection of ethylene using a gas chromatographic system has been presented by Janssen et al. in 2014. Parts per billion (ppb) by volume concentration was detected by adding the preconcentrator in the gas chromatographic system. The addition of preconcentrator results in a large humidity signal [5]. The use of a suitable stationary phase results in a separation of humidity from ethylene gas presented in an earlier work [12]. At a lower ppb concentration, drift in the baseline of a metal oxide gas sensor makes it difficult to measure the exact peak height of the chromatogram. The removal of the baseline drift using polynomial fit and the exact peak height determination of the chromatogram has been presented [13]. To operate the gas chromatographic system with synthetic air as a carrier gas, the metal oxide gas sensor shows a stable chromatogram. However, the use of ambient air as a carrier gas results in an uneven ethylene chromatogram. The electrochemical sensor eliminates the unevenness from the chromatogram when operated in ambient air.

Here, we report a gas chromatographic system with an electrochemical gas sensor that operates with ambient air. The use of ambient air makes the system practical for use in fruit containers, which is not feasible with synthetic air cylinders. The electrochemical gas sensor shows a similar response in ambient air and synthetic air. The complete system consists of a packed 3D printed gas chromatographic column with a heater, a preconcentrator, a mini-pump, a mass flow controller, and a humidity sensor. The use of a selective ethylene gas sensor along with the selective gas chromatographic column helps to avoid the uncertainty caused by ambient air, which disturbs the metal oxide gas sensor. 

The paper is structured as follows: Section 2 explains the metal oxide gas sensor and its limitations. Section 3 describes the use of the electrochemical ethylene gas sensor, its sensor electronics, and sensor calibration. Section 4 discloses the 3D printed gas chromatographic column with an attached heater. Section 5 briefly illustrates the preconcentrator. Section 6 reveals the controller to operate the gas chromatographic system. Section 7 depicts the system design. Section 8 reports that the system works. Section 9 discusses results, and Section 10 provides a conclusion and future work.

## 2. Metal Oxide Gas Sensors and Its Limitations

**Figure 1:**
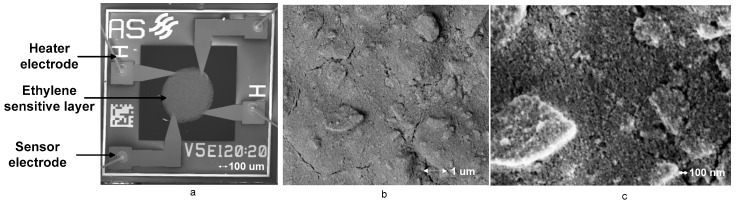
(**a**) SEM images of ASMLK gas sensor [14]; (**b**) a gas sensing layer; (**c**) a magnified image of the gas sensing layer.

In a previous work conducted for the detection of ethylene gas with a gas chromatographic system, a methane gas sensor was used (SnO_2_) [14]. Testing shows that the methane gas sensor shows good sensitivity towards ethylene. This is true because both are of a similar family of lower hydrocarbons. However, the metal oxide gas sensors change sensitivity when exposed to variable humidity. An increase in humidity results in the decrease in the sensitivity of the gas sensor. In one scenario, when the gas chromatographic system was placed in a certain environment, a change in the humidity of ambient air resulted in a change in the sensitivity of the sensor. This meant that system calibration was required before every measurement. The sensitivity of the metal oxide gas sensor is dependent on the sensitive layer. The SEM images of this sensor die are shown in Figure 1. The sensor consists of a gas sensitive layer and a heater wire bonded with four electrodes. The images of the sensor layer reveal that the sensing layer is porous (maximum 90 nm in diameter). Using the gas chromatographic system with ambient air as a carrier gas results in a decrease in the reduction of the surface area available for the adsorption of ethylene molecules and thus decreases the sensitivity of the sensor. A similar humidity effect on the metal oxide gas sensor is cited here [10,15,16].

## 3. Electrochemical Gas Sensor

A commercially available electrochemical ethylene gas sensor from Membrapor is used to detect ethylene gas is employed in the gas chromatographic system [17]. The range of the sensor to detect ethylene gas is 0–10 ppm with a resolution of 0.1 ppm. The electrochemical ethylene gas sensor is also sensitive to humidity and carbon monoxide as provided by the manufacturer. The electrochemical sensor contains an anti-condensation filter to filter dust and water vapor. The gas enters the sensor through three small capillaries. A hydrophobic filter is used to retain the electrolyte inside the plastic housing and to limit the effect of humidity. There are three electrodes present in the sensor, i.e., a sensing electrode, a reference electrode, and a counter electrode. The gas enters through a small capillary passing the anti-condensation filter and hydrophobic membrane by diffusion. The gas interacts with the sensing electrode where gas molecules oxidize or reduce, consequently producing or consuming electrons. The produced ionic current reaches the counter electrode by means of the electrolyte present in the sensor. 

**Figure 2:**
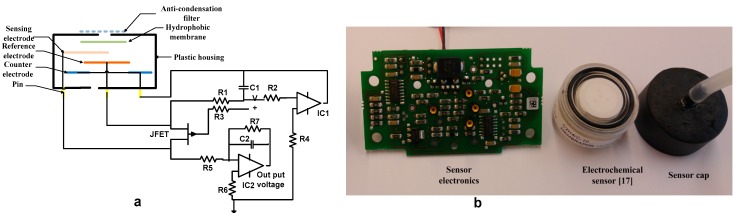
(**a**) Electrochemical sensor with sensor electronics; (**b**) Electrochemical gas sensor [17,18].

The electronics associated with the electrochemical is shown in Figure 2, which is a three-electrode potentiostatic circuit from Membrapor; see [18] for details on circuit operation. The op-amp IC1 is used to make measurements in a transimpedance configuration. Current is converted into voltage during chemical reaction with ethylene. The C2 is used to reduce high-frequency noises. The IC2 provides the current to the counter electrode to balance the current required by the sensing electrode. The use of the JFET makes sure the potential of the sensing electrode is the same as the reference electrode. 

**Figure 3:**
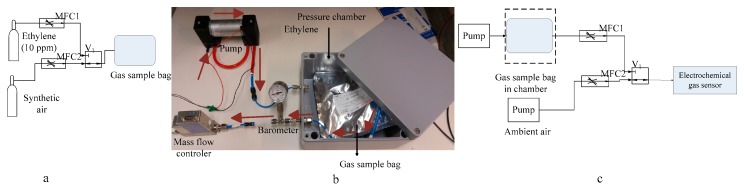
(**a**) Ethylene preparation in a gas sample bag; (**b**) the pump generates an excess pressure in the chamber, which squeezes the gas sample bag, and mass flow controller controls the ethylene gas flow; (**c**) the sensor calibration system.

To calibrate the sensor, commercially available 10 ppm ethylene gas is mixed with synthetic air in a gas sample bag to decrease the concentration. The gas sample bag is manufactured using a multilayer foil that has moisture protection [19]. The system used to fill the gas sample bag is shown in Figure 3a. The preparation of a 5 ppm ethylene concentration is done by mixing 0.5 L of 10 ppm ethylene gas with 0.5 L of synthetic air in a 1 L gas sample bag. The flow rate of both mass flow controllers are kept constant, and the time taken to switch between cylinders is recorded. Similarly, 5 ppm, 2.5 ppm, and 1.25 ppm ethylene gas is prepared in the same way. The bag is kept for 12 h for homogeneity and then used in the system.

The system of extracting ethylene from the gas sample bag is shown in Figure 3b, which consists of a pump, a pressure chamber with two ports, i.e., one port used to generate pressure in the chamber and another port used to take ethylene in the gas chromatographic system, a barometer, and a mass flow controller. When the pump is turned on, it draws air from the environment and discharges it into the chamber. This generates a positive pressure into the sealed chamber. As the pressure reaches 0.2 bar it squeezes the gas sample bag inside it and causes the ethylene to emerge from the gas sample bag. The flow of ethylene is controlled using the mass flow controller.

To calibrate the sensor, the system consists of ethylene gas bags that contain 10 ppm, 5 ppm, 2.5 ppm, and 1.25 ppm of ethylene gas, a solenoid valve, a mini-pump, and the electrochemical sensor shown in Figure 3c. Ambient air flows through the sensor at a flow rate of 30 L/min. It takes around 15 min to attain a stable baseline with ambient air. The two potentiometers on the circuit board are used to calibrate the sensor electronic together with the sensor. The potentiometer “zero” is adjusted to produce an output signal of 40 mV. We used a maximum gas concentration of 10 ppm. When the signal is stable; the potentiometer “span” was adjusted with the gain of 10 to produce an output signal of 2 V [18]. 

**Figure 4:**
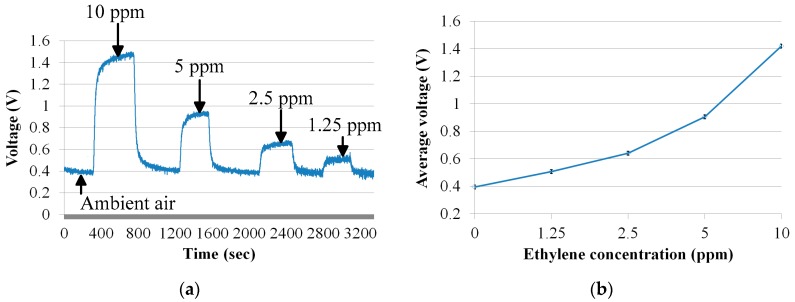
(**a**) Electrochemical sensor response at ambient air, 10 ppm, 5 ppm, 2.5 ppm, and 1.25 ppm; (**b**) the relationship between the concentration of ethylene and the output voltage.

After sensor calibration, ethylene gas concentrations of 10 ppm, 5 ppm, 2.5 ppm, and 1.25 ppm, as well as ambient air, are applied to the electrochemical gas sensor at a flow rate of 10 sccm. The response of the sensor is shown in Figure 4a. The relationship between the output voltage and ethylene concentration is shown in Figure 4b. 

## 4. Gas Chromatographic Column

**Figure 5:**
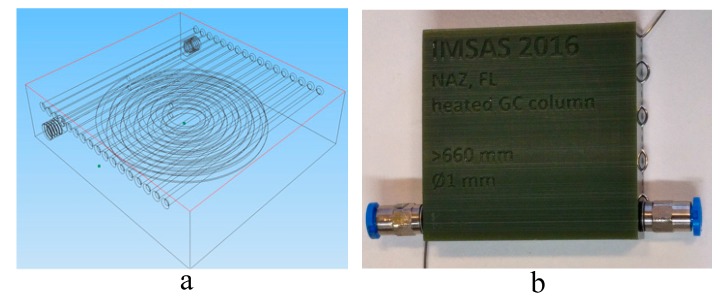
(**a**) Computer-aided design CAD gas chromatographic column diagram; (**b**) a printed column.

A 660 mm stacked spiral gas chromatographic column with a 1 mm column diameter is printed with integrated fluidic connectors as shown in Figure 5. The gas chromatographic column is printed using a high-resolution stereo-lithography printer (Perfactory Micro HiRes, EnvisionTEC Inc., Dearborn, MI, USA), and acrylic plastic (HTM140 M, EnvisionTEC Inc., Dearborn, MI, USA) is used as printing material. The design of the column is made in Inventor computer-aided design CAD software. The column is packed with a carbosieve SII stationary phase [20]. For integrated design, 18 straight channels are introduced into the design, as shown in Figure 5. The platinum metal wire is rolled into channels. For uniformity of the heating profile to the gas chromatographic column, all the holes are placed at equidistance. Fillets are added at the end of the channels in order to prevent wires from breaking. To continuously monitor the temperature, a commercially available PID (Proportional Integral and Derivative) temperature controller (CAL 3300, Cal Controls, Hitchin, UK) is used with the k-type sensor and solid state relay. 

## 5. Preconcentrator

**Figure 6:**
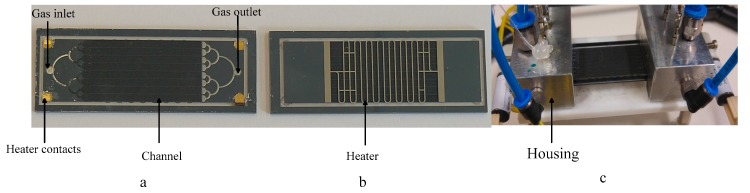
Preconcentrator. (**a**) Front side; (**b**) back side; (**c**) with housing.

The preconcentrator is fabricated in silicon technology, as shown in Figure 6. The preconcentrator consists of eight straight channels, a heater, heater contacts, and an inlet and outlet of gas. The dimensions of a single channel are 40.0 mm × 2.0 mm × 0.9 mm. The eight channels contain carbosieve SII [20]. The preconcentrator chip is placed in a housing to avoid gas leakage. The housing contains two pneumatic connectors, four push-in connectors, and two solenoid valves. The pneumatic connector avoids the outgassing. The push-in connectors provide voltage to the heater, and the solenoid valves pass or bypass the preconcentrator in the gas chromatographic system. The simulation that provides the heating profile and the fabrication steps are presented elsewhere [5].

## 6. Controller Design

The controller used to operate the complete system is the on–off controller. The algorithm of the controller was designed in LabVIEW. A National Instruments DAQ card is used as an on–off controller and for data acquisition. Labview version 2015 is installed on the Windows-operated tablet with an Intel Atom x5-Z8300 Processor (1.84 GHz, 2 MB Cache, SurfTab duo W1, TrekStor, Lorsch, Germany) and a RAM of 2 GB. The components that need to be controlled are the solenoid valves, the pump, the gas chromatographic column heater, and the preconcentrator heater. The data acquisition (DAQ) card cannot directly drive the components, so the transistor and relays are used to increase the power. The following are the main features of the controller.

- For data acquisition, a moving average filter of order 2 is used to minimize the noise present in the sensor.
- The heater attached to the gas chromatographic column is operated at 10 V and is continuously ON to keep the temperature at 45 °C.
- The heater attached with the preconcentrator is operated at 10 V—turned ON for 10 min and then turned off.
- The valves to switch between ethylene and air, bypass the preconcentrator, and bypass the gas chromatographic column are operated at 5 V and are switched ON and OFF according to Section 8.
- A graphical user interface (GUI), designed in Labview, presents a real-time data plot and controls all of the components attached to the gas chromatographic system.

When the system starts, a connection is built between the Labview and the DAQ card using software DAQmax. Followed by data acquisition executed by the program, the DAQ card and real-time plot can be seen on the tablet. Virtual buttons are implemented in the Labview program to control the switching preconcentrator, the gas chromatographic column, the heater, and the sensor. The data is stored with a time interval of 0.25 s. After the end of each experiment, sensor data is saved in an Excel file, which is plotted and further analyzed. 

## 7. System Design

**Figure 7:**
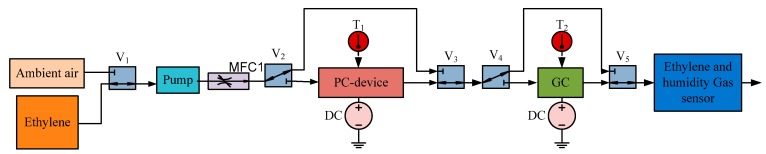
Gas chromatographic system.

**Figure 8:**
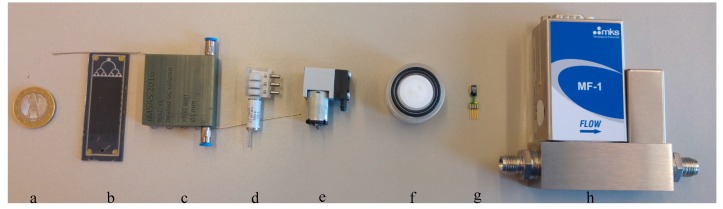
Individual components in the gas chromatographic system. (**a**) One euro (for size reference); (**b**) preconcentrator; (**c**) gas chromatographic column; (**d**) valve; (**e**) mini-pump; (**f**) electrochemical gas sensor; (**g**) humidity sensor; (**h**) mass flow controller.

The system consists of a mini-pump, solenoid valves, a preconcentrator, a gas chromatographic column, an electrochemical ethylene gas sensor, and a humidity sensor. The block diagram of the system is shown in Figure 7. The humidity sensor is SHT 75 from Sensirion [21]. The sensor is interfaced with the LabVIEW card using I^2^C communication [22]. The pump operates at 5 V with a flow rate of 500 sccm [23]. This flow rate is decreased by attaching a mass flow controller to attain a flow rate of 10 sccm. Solenoid valves V1 to V5 with a 3-Way Soft Tube Ported Style from Lee HDI-Ventile are added to switch between ambient air and ethylene gas, preconcentrator, and the gas chromatographic column [24]. The gas chromatographic column is heated using the PID controller. The 400 ppb ethylene gas (N30) and the synthetic air are purchased from Air Liquide [25,26]. The individual components are shown in Figure 8. The data from the sensors are collected in an NI DAQ 6212 data acquisition card connected to Labview on a personal computer [22]. 

## 8. The Working System

The five valves V_1_ to V_5_ are used for switching ethylene and air in the preconcentrator and in the gas chromatographic column. The preconcentrator traps the ethylene molecules within an 18 min period. This process is termed as adsorption and carried out at room temperature at a flow rate of 10 sccm. After adsorption, the preconcentrator is bypassed, and ambient air cleans the remaining ethylene in pneumatic channels. To attain the baseline, the ambient air is passed through the gas chromatographic column. A National Instrument data acquisition card (DAC) is interfaced between the sensor and the personal computer (PC). A graphical user interface (GUI) developed in Labview displays the baseline. A preconcentrator is heated for 250 °C for 10 min, which detaches the trapped ethylene molecules from the stationary phase used in the preconcentrator. Ambient air flows into the preconcentrator using the valves that take the detached ethylene molecules. The ethylene molecules enter into the gas chromatographic column where the ethylene gas is retained and separated from humidity. 

## 9. Result and Discussion

**Figure 9:**
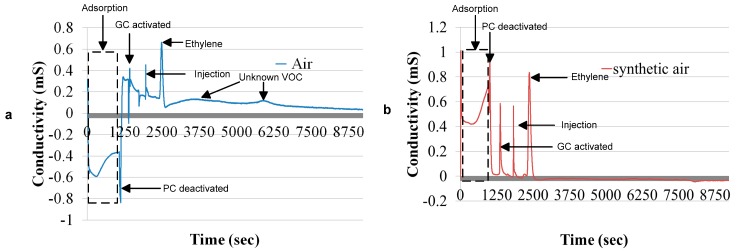
(**a**) 400 ppb with a metal oxide gas sensor and an ambient air carrier gas; (**b**) 400 ppb with a metal oxide gas sensor with a synthetic air carrier gas.

Figure 9a,b show the chromatogram from the metal oxide gas sensor with a probe gas of 400 ppb. A peak is obtained at 1213 s, as the preconcentrator is deactivated. At 1653 s, the gas chromatographic column is activated, which again produces another peak. These peaks occur because the metal oxide gas sensor is sensitive to the change in flow rate as the preconcentrator and the gas chromatographic column switch ON and OFF. The other peaks are the injection peak and the ethylene peak. The ethylene chromatogram arrives at 2750 s and lasts for 3080 s. 

Figure 9a signifies the chromatogram with ambient air as a carrier gas, and Figure 9b indicates the chromatogram with synthetic air as a carrier gas. A comparison between both the chromatogram reveals that the sensitivity of the sensor towards ethylene gas decreases as the peak height is reduced with ambient air. Furthermore, the bell shape of the chromatogram is distorted with ambient, air and uncertain peaks can also be seen in the chromatogram. A reduction in the sensitivity of the gas sensor is a result of the reduction in the surface area of the sensor for the adsorption of ethylene gas, as explained in Section 2. 

**Figure 10:**
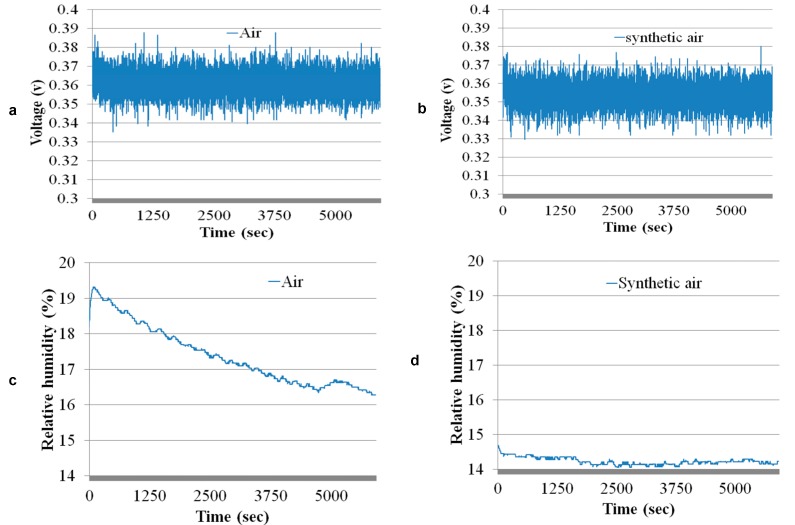
(**a**) Baseline of the electrochemical sensor with ambient air; (**b**) baseline of the electrochemical sensor with synthetic air; (**c**) baseline of the humidity sensor with air; (**d**) baseline of the humidity sensor with synthetic air.

Figure 10a,b show the similarity in the baselines from the electrochemical ethylene gas sensors with ambient air and with synthetic air. Both of these baselines are acquired while detecting the humidity from the humidity sensor. Figure 10c,d reveal that the humidity varies in ambient air compared to the synthetic air. This result is factual, as synthetic air is produced under precise conditions. However, the effect of the drift in the humidity cannot be seen in the baseline from the electrochemical sensor because the filter in that sensor suppresses the humidity. 

**Figure 11:**
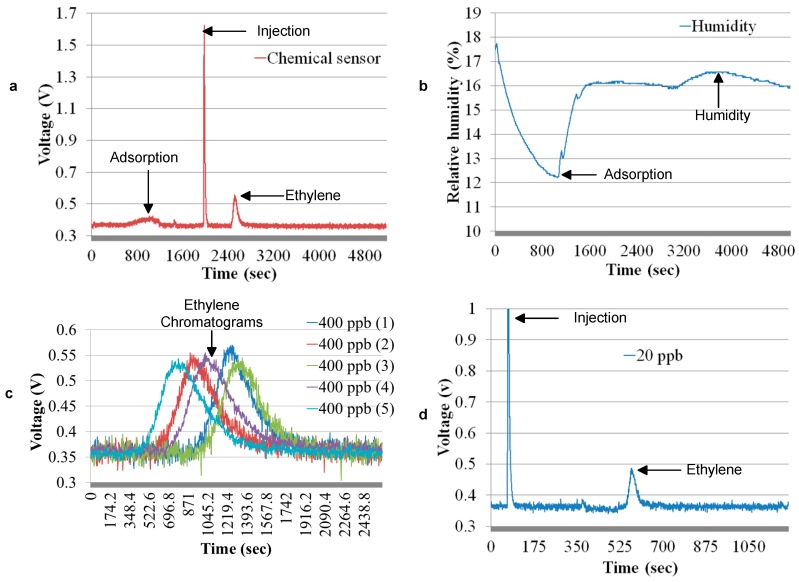
(**a**) 400 ppb with air as a carrier gas; (**b**) 400 ppb with air carrier gas with a humidity sensor; (**c**) sensor repeatability; (**d**) 20 ppb with air as a carrier gas.

Figure 11a shows a procedure of acquiring ethylene chromatogram from the system. The first 1080 s is preconcentration. In the first 480 s, the preconcentrator adsorbs ethylene. After adsorption, the preconcentrator is saturated as the sensor slowly starts detecting ethylene gas. During preconcentration, the humidity sensor exponentially decreased, which is also because the material insider the preconcentrator adsorbs the humidity [20]. 

In the chromatogram shown in Figure 11a, the first peak is the injection peak, which is at 1860 s. A 400 ppb ethylene chromatogram arrives from the injection peak after 480 s. The humidity arrives from the injection peak after 1080 s. The humidity sensor shows a curve, as shown in Figure 11b. The gas chromatographic column separates ethylene and humidity.

To measure the repeatability, five 400 ppb chromatograms were obtained under the same conditions, as shown in Figure 11c. Areas under the curve of the chromatograms are calculated using the Riemann sums method. An assessment of the results shows that the system is 1.2% repeatable. 

To further detect in tens of ppb, 400 ppb ethylene gas is mixed with synthetic air in a gas bag. An ethylene of 20 ppb concentration is prepared by mixing 0.05-L ethylene concentration of 400 ppb in 0.95-L synthetic air. Ethylene at 20 ppb was detected under the same conditions as described above. A chromatographic at 20 ppb ethylene is shown in Figure 11d. The non-linearity in the area of the chromatogram is because of the fact that the preconcentrator is more effective at lower concentrations, as compared to higher concentration [5]. 

To determine the resolution, we first calculated the standard deviation of the measurement noise, which is 0.7 ppb; thus, the minimum detection limit was 3σ = 2.3 ppb for the gas chromatographic system.

## 10. Conclusions and Future Work

A gas chromatographic system that consists of a mini-pump, a mass flow controller, a preconcentrator, a gas chromatographic column, a humidity sensor, and an electrochemical ethylene gas sensor is presented here. The sensitivity of a metal oxide gas sensor in a gas chromatographic system is influenced by the environmental humidity. The ambient air used as a carrier gas results in a decrease in the sensitivity of the gas sensor. To use the system with ambient air, an electrochemical ethylene gas sensor is employed in the system. A 660 mm stacked spiral chromatographic is designed and printed in a 3D printer. A platinum wire is wrapped around the column, heated using a PID controller and a k-type sensor. The flow rate of the ambient air is fixed to 10 sccm. The column separated the humidity and the ethylene gas. The results of the electrochemical ethylene gas sensor revealed no baseline drifts or uncertain peaks when operated with ambient air. The system was tested with 400 ppb and 20 ppb ethylene gas. The gas chromatographic system was able to detect ethylene with ambient air. The system’s minimum detection limit was 2.3 ppb.

In future work, the system should be studied at lower ppb concentrations, and the resolution should be further tested with different ethylene concentrations. Furthermore, the system will be further verified with ripening bananas stored in a box under several different gases. 

## References

[1] Gustavsson J., Cederberg C., Sonesson U., van Otterdijk R., Meybeck A. (2011). Global Food Losses and Food Waste. Extent, Causes and Prevention.

[2] Gong S., Liang H. (2006). On the logistics network modes of fresh food cold chain. China’s Econ. Circ..

[3] Janssen S., Schmitt K., Blanke M., Bauersfeld M.L., Wlenstein J., Lang W. (2014). Ethylene detection in fruit supply chains. Philos. Trans. R. Soc. A.

[4] Tanner D.J., Amos N.D. (2003). Heat and mass transfer—Temperature variability during shipment of fresh produce. Acta Hortic..

[5] Janssen S., Tessmann T., Lang W. (2014). High sensitive and selective ethylene measurement by using a large-capacity-on-chip preconcentrator device. Sens. Actuators B Chem..

[6] Saltveit M.E. (1999). Effect of ethylene on quality of fresh fruits and vegetables. Postharvest Biol. Technol..

[7] Blanke M.M. (2014). Reducing ethylene levels along the food supply chain: A key to reducing food waste?. J. Sci. Food Agric..

[8] Cristescu S.M., Mandon J., Arslanov D., Pessemier J.D., Hermans C., Harren F.J.M. (2013). Current methods for detecting ethylene in plants. Ann. Bot..

[9] Biasio M.D., Leitner R., Krall C., Krivec M., Wilk A., Mizaikoff B., Waldner R., Starmans F., Maier D. Ethylene gas sensing using non-dispersive infrared spectroscopy. Proceedings of the IEEE SENSORS.

[10] Krivec M., Gunnigle G.M., Abram A., Maier D., Waldner R., Gostner J.M., Überall F., Leitner R. (2015). Quantitative Ethylene Measurements with MOx Chemiresistive Sensors at Different Relative Air Humidities. Sensors.

[11] Besar K., Dailey J., Katz H.E. (2017). Ethylene Detection Based on Organic Field-Effect Transistors with Porogen and Palladium Particle Receptor Enhancements. ACS Appl. Mater. Interfaces.

[12] Lucklum F., Janssen S., Lang W., Vellekoop M.J. Miniature 3D gas chromatography columns with integrated fluidic connectors using high-resolution stereolithography fabrication. Proceedings of the Eurosensors.

[13] Zaidi N.A., Tahir M.W., Vinayaka P.P., Lucklum F., Vellekoop M., Lang W. Detection of ethylene using gas chromatographic system. Proceedings of the Eurosensors 2016.

[14] Natural Gas Sensor. http://www.gassensor.ru/data/files/combustible-gas/CH4_AK01.pdf.

[15] Kuang Q., Lao C., Wang Z.L., Xie Z., Zheng L. (2007). High-sensitivity humidity sensor based on a single SnO_2_ nanowire. J. Am. Chem. Soc..

[16] Bârsan N., Weimar U. (2013). Understanding the fundamental principles of metal oxide based gas sensors; the example of CO sensing with SnO_2_ sensors in the presence of humidity. J. Phys. Condens. Matter.

[17] Electrochemical Gas Sensor. http://www.membrapor.ch/sheet/C2H4-C-10.pdf.

[18] Application Note MEM1 Electrochemical Gas Sensor. http://www.membrapor.ch/sheet/Transmitter%20Compact.pdf.

[19] Sigma-Aldrich Supel™-Inert Multi-Layer Foil. Volume 1 L, Screw Cap Valve (SCV) Bag. http://www.sigmaaldrich.com/catalog/product/supelco/30226u?lang=de&region=DE&cm_sp=Insite-_-prodRecCold_xviews-_-prodRecCold10-1.

[20] Sigma-Aldrich (2014). “Carbosieve Adsorbent”, Matrix Carbosieve SII, 80–100 Datasheet. https://www.sigmaaldrich.com/content/dam/sigma-aldrich/docs/Supelco/Bulletin/t195890.pdf.

[21] Sensirion Humidity and Temperature Sensor IC. https://www.sensirion.com/fileadmin/user_upload/customers/sensirion/Dokumente/2_Humidity_Sensors/Sensirion_Humidity_Sensors_SHT7x_Datasheet_V5.pdf.

[22] DAQ M Series, NI USB-621x User Manual. https://www.ni.com/pdf/manuals/371931f.pdf.

[23] Gas and Air Pump, Eccentric Diaphragm Pump EC Serie 200 EC-LC. http://www.schwarzer.com/pages_en/produkt.php?id=50.

[24] LHD Series Solenoid Valves. http://www.theleeco.com/electro-fluidic-systems/solenoid-valves/lhd/lhd-series-solenoid-valves.cfm.

[25] Ethen N_30_, Air Liquid. http://produkte.airliquide.de/gasekatalog/pdb/ethenn30.pdf.

[26] Synthetic Air, ALPHAGAZ™ 1 LUFT. http://produkte.airliquide.de/gasekatalog/pdb/alphagaz1luft.pdf.

